# Prevalence of Primary Dysmenorrhea and Its Effect on the Quality of Life Among Female Students at Umm Al-Qura University

**DOI:** 10.7759/cureus.72136

**Published:** 2024-10-22

**Authors:** Yasir Katib, Mariah Almehmadi, Fatima Alhajaji, Salihah Alqorashi, Fathia Almajnooni, Marwan A Alshinawi, Renad Ahmed Marghalani

**Affiliations:** 1 Department of Obstetrics and Gynecology, Umm Al-Qura University, Makkah, SAU; 2 Faculty of Medicine, Umm Al-Qura University, Makkah, SAU; 3 Faculty of Medicine, Ibn Sina National College for Medical Studies, Jeddah, SAU

**Keywords:** academic performance, dysmenorrhea, menstrual pain, menstruation, pain intensity, pelvic pain, premenstrual syndrome, quality of life, saudi arabia

## Abstract

Introduction

Primary dysmenorrhea is a common menstrual disorder causing significant physical and emotional discomfort, particularly among young women. This study aimed to determine the prevalence of primary dysmenorrhea, its associated symptoms, and its impact on the quality of life among female students at Umm Al-Qura University in Saudi Arabia.

Methodology

A cross-sectional study was conducted among 620 female students aged 18-30 using a validated, self-reported questionnaire. Data collected included demographic information, menstrual history, dysmenorrhea characteristics, and quality of life measured by the Short Form Health Survey-12 (SF-12). The confidence interval (CI) was maintained at 95%, and a p-value of <0.05 was selected as the statistically significant level in all tests. Statistical analysis was performed using IBM SPSS Statistics for Windows, Version 27 (Released 2020; IBM Corp., Armonk, New York).

Results

The prevalence of primary dysmenorrhea was 83.7%. Of the participants, 51.1% reported moderate pain, while 32.6% experienced severe pain (pain score 8-10). Premenstrual syndrome was reported by 71.8%. Pain significantly impacted physical functioning, with 75.5% reporting decreased productivity due to menstrual pain, and 76.3% noted emotional problems. Family history of menstrual pain was present in 53.2% of the participants, and BMI had no significant correlation with pain severity.

Conclusions

Primary dysmenorrhea significantly affects the quality of life and academic performance of female students. Effective management strategies and increased awareness are essential to reduce its impact on young women's lives.

## Introduction

Dysmenorrhea is characterized by painful cramps during menstruation that arise from the uterus. Dysmenorrhea is generally categorized into two types: primary dysmenorrhea, which involves pain without an underlying organic cause, and secondary dysmenorrhea, which is associated with specific pathological conditions such as endometriosis or ovarian cysts [1]. Despite its widespread occurrence, dysmenorrhea remains poorly understood and is often overlooked when monitoring women's health [2]. Symptoms such as lower abdominal pain or cramping are frequently accompanied by headaches, dizziness, diarrhea, bloating, nausea, vomiting, back pain, and leg discomfort [3,4]. The majority of research on primary dysmenorrhea has primarily involved students and adolescents, which restricts the relevance of the findings for women across all adult age groups, including middle-aged women. As a result, additional clarification is necessary [5]. Moreover, the prevalence of primary dysmenorrhea is probably significantly underestimated, as many women tend to link it with regular menstrual pain and do not seek medical care for this illness [6].

Dysmenorrhea is the most common symptom associated with menstrual issues and contributes to more illness in developing nations than any other gynecological condition [7]. Globally, millions of women experience the effects of primary dysmenorrhea. According to recent local studies, dysmenorrhea affects more than two-thirds of Saudi females [8,9]. Students find it challenging to maintain an acceptable level of academic performance during menstruation. Several studies have found that significant menstrual pain is related to absences from school or work, as well as difficulties in performing daily tasks [10,11].

Menstruation plays a crucial role in the assessment of reproductive and endocrine health in women [12,13]. Although it is a natural process, many girls experience menstrual issues such as irregular cycles, heavy bleeding, and painful periods [14]. Numerous studies have examined the prevalence of dysmenorrhea in relation to various factors, such as smoking, diet, physical inactivity, body mass index (BMI), caffeine intake, early onset of menarche, and psychological and genetic influences, among others [15,16]. Changes in lifestyle, such as eating a low-fat diet, such as the vegetarian diet, were found to reduce the frequency and severity of menstrual cramps [17]. However, it remains uncertain which of these factors has the most significant impact on dysmenorrhea in women.

The aim of this study is to determine the prevalence of primary dysmenorrhea and the features associated with it among female students at Umm Al-Qura University, Makkah, Saudi Arabia. In addition, this study aims to assess its impact on quality of life to help students recognize ways to mitigate the severity of their symptoms, encouraging them to make the necessary changes to improve their quality of life.

## Materials and methods

Study design

A descriptive, cross-sectional study using a convenience sampling technique was conducted at Umm Al-Qura University to assess the prevalence of primary dysmenorrhea and its effect on the quality of life among female students aged between 18 and 30 years who are pursuing a bachelor's degree at Umm Al-Qura University. Postgraduate female students, students with known pelvic pathologies, and those who refused to participate in this survey were excluded from the study.

Sample size

The minimum sample size required for this study was calculated by OpenEpi version 3.0, considering the following: the target population for our study is approximately 50,000 people, keeping the confidence interval (CI) level at 95%, considering the anticipated % of frequency as 50%, and taking the design effect as 1. Therefore, the sample size was calculated to be 384 participants. In case of any possible data loss, we plan to maximize the total sample size to reach 400 participants.

Data collection

A validated questionnaire from a previous study [18] was distributed using Google Forms via social media apps, revised, and translated into Arabic by a validated translator using a back-to-back technique. The questionnaire comprised four main components. The first section asked the participants about their demographic data, including age, nationality, marital status, BMI, college they studied, GPA, and academic year. The second section is about menstruation history, including age at menarche, regularity of menstrual cycle, duration of cycle, and flow. The third section assessed the factors associated with dysmenorrhea, including the onset, duration, and intensity of pain, associated symptoms, and methods used to reduce pain. The fourth section assessed the impact of menstrual pain on the quality of life of students, which was monitored using the Short Form Health Survey-12 (SF-12) [19]. The final section covers the impact of dysmenorrhea on academic performance and how it affects students. A pilot study was conducted with 20 female students to assess the reliability and validity of the questionnaire and to measure the time required for completion. To enhance clarity and understanding, complex medical terminology was omitted.

Statistical analysis

Descriptive Statistics

Descriptive statistics for the SF-12 scores were computed to summarize the central tendency and dispersion of the data. This included the mean, standard deviation (SD), median, and interquartile range (IQR) for each domain of the SF-12. The mean provides the average score, while the SD indicates the variability around the mean. The median represents the middle value of the data, and the IQR reflects the range within which the central 50% of scores lie.

Scoring Systems

The SF-12 assesses health-related quality of life across various domains. It includes physical functioning, role limitations due to physical health, role limitations due to emotional problems, energy/fatigue, emotional well-being, social functioning, pain, and general health perception. Scores on the SF-12 range from 0 to 100, with higher scores reflecting better health-related quality of life.

Inferential Statistics

Inferential statistics were used to determine the significance of differences and relationships within the data. For comparing SF-12 scores between groups, the Mann-Whitney U test was employed for non-normally distributed variables. Spearman's rank correlation coefficients were calculated to assess the strength and direction of associations between BMI and SF-12 domain scores.

Significance

Statistical significance was determined at a threshold of p < 0.05. Results with p-values less than this level were considered significant, indicating that the observed effects or differences were unlikely to have occurred by chance.

Software

Data analysis was performed using IBM SPSS Statistics for Windows, Version 27 (Released 2020; IBM Corp., Armonk, New York). This software was utilized for all statistical computations, including descriptive statistics, inferential tests, and correlation analyses.

Ethical consideration

The research proposal received ethical approval from the Umm Al-Qura University Institutional Research Board (IRB) (approval no. HAPO-02-K-012-2024-06-2173). The questionnaire began with a clear statement assuring participants that their information would remain anonymous and used strictly for research reasons. Participants were asked to agree or deny participation, and only those who agreed were included in the study. To ensure confidentiality and anonymity, each research participant was allocated a unique code number that was only utilized for data analysis. Participation in the study was entirely voluntary, with no incentives given. Furthermore, participants' names were protected in any published studies.

## Results

Demographic, academic, and menstrual health characteristics of the study participants

The study population comprised 620 participants, the majority aged between 18 and 20 years (N=339, 54.7%), followed by those aged 20-22 years (N=166, 26.8%). Most were single (N=591, 95.3%). Participants were primarily from medical colleges (N=232, 37.4%), followed by faculties of Sharia and Administration (N=172, 27.7%), Science and Engineering (N=126, 20.3%), and Humanities and Education (N=90, 14.5%). Over half of the participants had a cumulative GPA between 3.5 and 4.0 (N=381, 61.5%), while 25.6% (N=159) had a GPA between 3.0 and 3.49. The distribution of academic years showed that most were in their first year (N=147, 23.7%) or second year (N=125, 20.2%). The average weight was 55.1 kg (SD=14.4), the average height was 157.6 cm (SD=7.1), and the mean BMI was 22.19 (SD=6.22). Most participants had their first period between the ages of 11 and 14 (N=480, 77.4%) and reported regular menstrual cycles (N=440, 71.0%). The majority experienced menstrual bleeding lasting two to eight days (N=534, 86.1%), with normal blood loss (N=477, 76.9%). A high proportion reported premenstrual syndrome symptoms with every menstrual cycle (N=445, 71.8%). Slightly more than half (N=330, 53.2%) had a family history of menstrual pain, primarily in first-degree relatives (N=270, 81.8%) (Table 1).

**Table 1:** Demographic, academic, and menstrual health characteristics of the study participants n: number; %: percentage; SD: standard deviation

		N	%
Age	18–20	339	54.7%
	20–22	166	26.8%
	23–25	104	16.8%
	25–28	6	1.0%
	>30	5	0.8%
Marital status	Single	591	95.3%
	Married	27	4.4%
	Divorced/widow	2	0.3%
University department	Colleges of Science and Engineering	126	20.3%
	Faculties of Humanities and Education	90	14.5%
	Faculties of Sharia and Administration	172	27.7%
	Medical colleges	232	37.4%
Cumulative GPA	<2	6	1.0%
	2–2.49	20	3.2%
	2.5–2.9	54	8.7%
	3–3.49	159	25.6%
	3.5–4	381	61.5%
Academic year	First year	147	23.7%
	Second year	125	20.2%
	Third year	119	19.2%
	Fourth year	92	14.8%
	Fifth year	59	9.5%
	Sixth year	29	4.7%
	Seventh year	49	7.9%
Weight (kg) mean (±SD)	55.1 (±14.4)		
Length (cm) mean (±SD)	157.6 (±7.1)		
BMI (kg/m^2^) mean (±SD)	22.19 (±6.22)		
Your age when you had your first period (year)	≤10	65	10.5%
	11–14	480	77.4%
	≥15	75	12.1%
How long is the menstrual cycle between each period (days)?	Irregular (day <21 or day >35)	180	29.0%
	Regular (21–35 days)	440	71.0%
How long does menstrual bleeding last (days)?	Less than two days	46	7.4%
	2–8 days	534	86.1%
	Days >8	40	6.5%
How do you assess the amount of blood lost during menstruation?	Light bleeding (<7 pads used)	58	9.4%
	Normal bleeding (7–10 pads used)	477	76.9%
	Heavy bleeding (>10 pads used)	85	13.7%
Do you have premenstrual syndrome (mood changes a few days before your period, breast tenderness, appetite changes, fatigue, bloating, acne)?	No	10	1.6%
	Occasional	165	26.6%
	Yes, every menstrual cycle	445	71.8%
Do you have a family history of menstrual pain?	No	290	46.8%
	Yes	330	53.2%
If your answer is yes, what is the quantitative degree? (N=330)	First degree (mother, sisters)	270	81.8%
	Second degree (grandmothers, aunts, cousins)	60	18.2%

Menstrual pain characteristics, symptomatology, and lifestyle factors

Among the study participants, 51.1% (N=317) reported experiencing average pain (rated 4-7), while 32.6% (N=202) described their pain as intense (rated 8-10). Menstrual pain began from the first menstrual period for 25.6% (N=159), while 24.8% (N=154) experienced it one to three years after their first menstruation. Pain typically started on the first day of menstruation for 45.0% (N=279), with 59.7% (N=370) taking painkillers, of whom 48.6% (N=180) used medication once over the first three days. In terms of symptoms, fatigue was the most common (N=500, 80.6%), followed by nausea (N=356, 57.4%) and loss of appetite (N=354, 57.1%). Stress levels were high, with 34.2% (N=212) feeling stressed often. Regarding lifestyle habits, 52.9% (N=328) did not consume fruits or vegetables, and 69.5% (N=431) consumed caffeine. Sleep patterns showed that 47.9% (N=297) slept more than seven hours a day, as shown in Table 2.

**Table 2:** Menstrual pain characteristics, symptomatology, and lifestyle factors

		N	%
How severe is the pain?	Light 1–3	101	16.3%
	Average 4–7	317	51.1%
	Intense 8–10	202	32.6%
When did the menstrual pain start?	From the first menstrual period	159	25.6%
	Less than a year	70	11.3%
	6–24 months after first menstruation	142	22.9%
	From 1–3 years	154	24.8%
	4 years and above	95	15.3%
When does menstrual pain start?	On the first day of menstruation	279	45.0%
	The day before menstruation	124	20.0%
	More than two days before menstruation	217	35.0%
Do you take painkillers for menstrual pain?	No	250	40.3%
	Yes	370	59.7%
If yes, how often do you use the medication in the first three days of menstruation? (N=370)	2 times/3 days	124	33.5%
	3 times/3 days	66	17.8%
	Once/3 days	180	48.6%
Does your menstrual pattern change when you take painkillers?	No	409	66.0%
	Yes	211	34.0%
Along with pain, what other symptoms occur during menstruation?	Fatigue	500	80.6%
	Nausea	356	57.4%
	loss of appetite for food	354	57.1%
	Arthralgia	342	55.2%
	Sweating	341	55.0%
	Headache	309	49.8%
	Feeling dizzy	285	46.0%
	Insomnia	235	37.9%
	Diarrhea	201	32.4%
	Vomiting	167	26.9%
	Agitation/irritability	123	19.8%
	Polyuria	92	14.8%
	Only the pain appears	90	14.5%
What is your stress level?	Usually (daily)	163	26.3%
	Sometimes (twice a week)	165	26.6%
	Often (3 times a week)	212	34.2%
	Hardly at all (once a month)	80	12.9%
Do you consume fruits/vegetables?	No	328	52.9%
	Yes	292	47.1%
Do you smoke?	No	589	95.0%
	Yes	31	5.0%
Do you consume caffeine?	No	189	30.5%
	Yes	431	69.5%
How many hours do you sleep a day? (hour)	≤4	30	4.8%
	5–7	293	47.3%
	>7	297	47.9%

Impact of menstrual health on daily activities, emotional well-being, and social functioning

In general, participants rated their health as very good (35.0%, N=217) or good (29.7%, N=184), while only 4.7% (N=29) described it as bad. During their last menstrual period, 45.3% (N=281) felt that moderate activities were somewhat restricted by their health, and 26.1% (N=162) felt significantly prevented from performing these activities. Climbing stairs was not an issue for 38.4% (N=238), yet 42.3% (N=262) experienced moderate restrictions. Regarding work and daily activities, 75.5% (N=468) felt they accomplished less than desired due to physical health, and 76.3% (N=473) reported emotional problems affecting their ability to work. Pain interfered with normal functioning to varying degrees for 92.8% (N=573). Feelings of sadness were reported all the time by 20.2% (N=125) (Table 3).

**Table 3:** Impact of menstrual health on daily activities, emotional well-being, and social functioning

			N	%
In general, would you describe your health		Bad	29	4.7%
		Excellent	91	14.7%
		Good	184	29.7%
		Moderate	99	16.0%
		very good	217	35.0%
The following questions are about activities you might do during your last period. Is your health likely to prevent you from doing these activities? If so, to what extent?	Moderate activities, such as moving a table, pushing a vacuum, bowling, or playing sports	No, it is not forbidden at all.	177	28.5%
		Yes, it prevents me a lot.	162	26.1%
		Yes, it's holding me back a little.	281	45.3%
	Climbing several flights of stairs	No, it is not forbidden at all.	238	38.4%
		Yes, it prevents me a lot.	120	19.4%
		Yes, it's holding me back a little.	262	42.3%
During your last menstrual period, did you experience any of the following problems with your work or other normal daily activities? As a result of your physical health?	I accomplish less than I want to	No	152	24.5%
		Yes	468	75.5%
	Were limited in type of work or other activities	No	179	28.9%
		Yes	441	71.1%
During your last menstrual period, did you experience any of the following problems with your work or other normal daily activities as a result of any emotional problems (such as feeling depressed or anxious)?	Accomplish less than you want	No	147	23.7%
		Yes	473	76.3%
	You did not do your work or other activities as carefully as usual	No	177	28.5%
		Yes	443	71.5%
During your last menstrual period, how much did the pain interfere with your normal functioning (including work outside the home and housework)?		It does not interfere at all	47	7.6%
		Moderately overlapping	119	19.2%
		Overlaps a little	183	29.5%
		Overlaps somewhat	167	26.9%
		overlaps to the maximum extent	104	16.8%
These questions are about how you feel and what things were like during your last period. For each question, please give the one answer that comes closest to how you felt.	Do you feel calm and peaceful?	All the time	54	8.7%
		Most of the time	109	17.6%
		A lot of time	71	11.5%
		Some time	219	35.3%
		Little time	124	20.0%
		None of the time	43	6.9%
	Do you have a lot of energy	All the time	27	4.4%
		Most of the time	62	10.0%
		A lot of time	67	10.8%
		Some time	168	27.1%
		Little time	178	28.7%
		None of the time	118	19.0%
	Are you feeling down and sad	All the time	125	20.2%
		Most of the time	175	28.2%
		A lot of time	108	17.4%
		Some time	120	19.4%
		Little time	71	11.5%
		None of the time	21	3.4%
	How long did your health or emotional problems interfere with your social activities (such as visiting friends, relatives, etc.)	All the time	164	26.5%
		Most of the time	147	23.7%
		A lot of time	106	17.1%
		Some time	108	17.4%
		Little time	59	9.5%
		None of the time	36	5.8%

Descriptive statistics for SF-12 health survey scores

**Figure 1:**
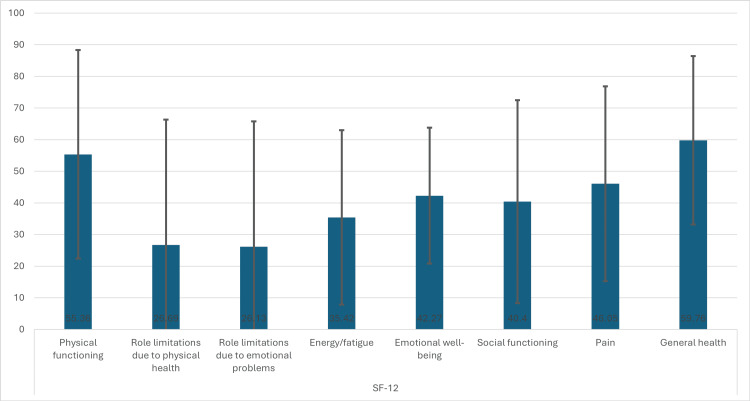
Health outcomes assessment: mean SF-12 scores

The mean score for physical functioning is 55.36 (SD=32.94), with a median of 50.00 and an interquartile range (IQR) of 25.00-75.00, indicating substantial variability in physical functioning. Role limitations due to physical health and emotional problems both have means around 26.69 (SD=39.60) and 26.13 (SD=39.62), respectively, with medians of 0 and IQRs extending up to 50.00, suggesting that many individuals experience significant limitations. The mean score for energy/fatigue is 35.42 (SD=27.56). Emotional well-being has a mean of 42.27 (SD=21.48). Social functioning scores have a mean of 40.40 (SD=32.10). Pain scores average 46.05 (SD=30.78) with a median of 50.00 and an IQR of 25.00-75.00, while general health has a higher mean of 59.76 (SD=26.61), with a median of 50.00 and an IQR of 50.00-75.00, indicating relatively better general health perceptions among participants (Table 4, Figure 1)

**Table 4:** Descriptive statistics for SF-12 health survey scores SD: standard deviation; IQR: interquartile range

	Mean	SD	Median	IQR
Physical functioning	55.36	32.94	50.00	25.00–75.00
Role limitations due to physical health	26.69	39.60	0.00	0.00–50.00
Role limitations due to emotional problems	26.13	39.62	0.00	0.00–50.00
Energy/fatigue	35.42	27.56	40.00	20.00–60.00
Emotional well-being	42.27	21.48	40.00	30.00–60.00
Social functioning	40.40	32.10	25.00	0.00–75.00
Pain	46.05	30.78	50.00	25.00–75.00
General health	59.76	26.61	50.00	50.00–75.00

Spearman's rank correlation between BMI and SF-12 health survey dimensions

The correlations for physical functioning, role limitations due to physical health, role limitations due to emotional problems, energy/fatigue, emotional well-being, and social functioning are all weak and not statistically significant. Specifically, the correlation coefficients range from -0.051 to 0.067, indicating minimal to no association between BMI and these dimensions of health. The only correlations approaching significance are for pain (ρ = 0.058, p = 0.147) and general health (ρ = 0.067, p = 0.097), which are still not significant, suggesting that BMI does not have a strong or meaningful impact on these aspects of health (Table 5).

**Table 5:** Spearman's rank correlation between BMI and SF-12 health survey dimensions **Correlation is significant at the 0.01 level (two-tailed) BMI: body mass index

		BMI
Physical functioning	Correlation coefficient	-.051
	Sig. (two-tailed)	.202
	N	620
Role limitations due to physical health	Correlation coefficient	.044
	Sig. (two-tailed)	.274
	N	620
Role limitations due to emotional problems	Correlation coefficient	-.045
	Sig. (two-tailed)	.265
	N	620
Energy/fatigue	Correlation coefficient	-.030
	Sig. (two-tailed)	.452
	N	620
Emotional well-being	Correlation coefficient	-.037
	Sig. (two-tailed)	.363
	N	620
Social functioning	Correlation coefficient	.018
	Sig. (two-tailed)	.660
	N	620
Pain	Correlation coefficient	.058
	Sig. (two-tailed)	.147
	N	620
General health	Correlation coefficient	.067
	Sig. (two-tailed)	.097
	N	620

Comparison of SF-12 health survey scores by family history of menstrual pain

The table provides results from an independent samples Mann-Whitney U test assessing differences in SF-12 health survey scores based on the presence of a family history of menstrual pain. For physical functioning, individuals with a family history of menstrual pain had a lower median score (50.00, IQR: 25.00-75.00) compared to those without (50.00, IQR: 50.00-100.00), with a significant p-value of <0.001. The scores for pain were also significantly different, with a median of 50.00 (IQR: 25.00-75.00) for those with a family history compared to the same median (IQR: 25.00-75.00) for those without, with a p-value of 0.039. Other dimensions, such as role limitations due to physical health, emotional problems, energy/fatigue, emotional well-being, social functioning, and general health, showed no significant differences between those with and without a family history of menstrual pain, as indicated by p-values greater than 0.05 (Table 6).

**Table 6:** Comparison of SF-12 health survey scores by family history of menstrual pain ^U^Independent samples Mann-Whitney U test *p<0.05, significant IQR: interquartile range

	Do you have a family history of menstrual pain?				
	No		Yes		p-value^U^
	Median	IQR	Median	IQR	
Physical functioning	50.00	50.00–100.00	50.00	25.00–75.00	<0.001*
Role limitations due to physical health	.00	0.00–50.00	0.00	0.00–50.00	0.125
Role limitations due to emotional problems	.00	0.00–50.00	0.00	0.00–50.00	0.178
Energy/fatigue	40.00	20.00–60.00	40.00	20.00–40.00	0.263
Emotional well-being	40.00	30.00–60.00	40.00	30.00–60.00	0.464
Social functioning	50.00	0.00–75.00	25.00	0.00–75.00	0.310
Pain	50.00	25.00–75.00	50.00	25.00–75.00	0.039*
General health	50.00	50.00–75.00	75.00	50.00–75.00	0.747

## Discussion

Primary dysmenorrhea significantly affects the quality of life of many young women. This study aimed to determine the prevalence of primary dysmenorrhea and its associated features among female students at Umm Al-Qura University, as well as to assess its impact on their quality of life.

The study revealed that over half of the participants experienced moderate menstrual pain, with 32.6% reporting severe pain (pain score 8-10). These findings align with those of previous studies conducted in Saudi Arabia, which have reported similar or higher rates of dysmenorrhea among female students [20]. A study among university students in Saudi Arabia found that 80.1% of participants suffered from moderate to severe dysmenorrhea [21]. The high prevalence of severe pain reveals the need for increased awareness and better pain management strategies to reduce the impact of dysmenorrhea on the daily lives of students.

Primary dysmenorrhea had a significant impact on the participants' quality of life, particularly on physical functioning and daily activities. More than 75% of participants reported that physical health and pain limited their ability to perform daily tasks. This is consistent with findings from other studies, which have highlighted the debilitating effects of dysmenorrhea on women's physical health and productivity. A study conducted in Spain among 299 students similarly found that dysmenorrhea led to absenteeism and reduced academic performance due to pain and physical discomfort [10].

In terms of emotional well-being, our study found that more than 70% of participants reported emotional disturbances, such as stress, sadness, and irritability, during their menstrual periods. These emotional impacts were significant and aligned with other studies that have explored the psychological effects of dysmenorrhea. Research indicates that women with primary dysmenorrhea are more likely to experience mood disturbances, anxiety, and depression during menstruation [22,23]. This highlights the importance of addressing not only the physical but also the emotional consequences of dysmenorrhea to improve the overall well-being of affected individuals.

Interestingly, the study did not find a strong correlation between BMI and the severity of dysmenorrhea or its impact on health-related quality of life. Previous research on the relationship between BMI and dysmenorrhea has shown mixed results, with some studies suggesting a positive association between higher BMI and increased menstrual pain [24]. However, other studies, like ours, have found no significant correlation [25]. This suggests that BMI may not be a consistent predictor of dysmenorrhea severity, and more research is needed to explore other potential factors, such as hormonal levels and lifestyle habits, that may contribute to menstrual pain.

This study also revealed that 53.2% of participants had a family history of menstrual pain, and those with a family history reported significantly lower scores in physical functioning and higher pain levels. This is in line with previous studies that have identified a genetic predisposition to dysmenorrhea, with first-degree relatives often experiencing similar menstrual pain [26]. A study in Ethiopia similarly found that a family history of dysmenorrhea was a strong predictor of the condition's severity and recurrence [27]. It suggests that genetic factors play a role in the development of primary dysmenorrhea and should be considered when evaluating patients for appropriate management strategies.

Lifestyle factors, such as dietary habits and caffeine consumption, were also examined in this study. We found that more than half of the participants did not consume fruits and vegetables regularly, and 69.5% consumed caffeine. Previous research has suggested that poor dietary habits and high caffeine intake may exacerbate menstrual pain. A study in Saint Vincent and Grenadines found that a diet low in fruits and vegetables was associated with an increased risk of dysmenorrhea, while high caffeine consumption was linked to higher pain intensity [28]. It supports the need for educational interventions that promote healthier dietary habits to potentially alleviate menstrual symptoms.

The widespread use of painkillers among participants, with nearly 60% using medication to manage their pain, particularly non-steroidal anti-inflammatory drugs (NSAIDs). The frequent use of painkillers is consistent with global trends, where NSAIDs are commonly used as the first line of treatment for dysmenorrhea [29,30]. However, while these medications provide temporary relief, they do not address the underlying causes of dysmenorrhea. Long-term reliance on painkillers can also lead to side effects, underscoring the need for alternative management strategies, such as lifestyle modifications, exercise, and stress reduction techniques [31].

Regarding the impact of dysmenorrhea on academic performance, 75.5% of participants reported that menstrual pain interfered with their ability to concentrate and complete academic tasks. This finding is consistent with research conducted in various countries, which has demonstrated that dysmenorrhea is a leading cause of school absenteeism and decreased academic performance among female students. A study conducted in Eastern Ethiopia reported similar findings, with dysmenorrhea significantly affecting the academic performance and social activities of adolescent girls [32]. It emphasizes the importance of providing adequate support to students dealing with menstrual pain to ensure their academic success.

The study has important implications for health education and the development of interventions aimed at improving the management of dysmenorrhea. Given the high prevalence and significant impact of dysmenorrhea on physical, emotional, and academic well-being, there is a clear need for increased awareness and access to effective treatment options. Educational programs that teach students about menstrual health, pain management techniques, and the importance of a healthy lifestyle could help mitigate the effects of dysmenorrhea and improve the quality of life of those affected.

Limitations

This study had a few limitations. It was based on self-reported data from students, which means that the accuracy of the information relies on the participants' memory and honesty. Some may have over- or under-reported their symptoms or lifestyle habits. It focused only on students from one university, so the findings may not apply to all women in different regions or age groups. We did not explore other possible causes of menstrual pain, such as underlying health conditions, which could have affected the results.

Future research

Future research should look at larger and more diverse groups of women, including those from different universities or age ranges, to get a broader understanding of how primary dysmenorrhea affects different populations. It would also be helpful to study the long-term impact of lifestyle changes, such as diet and exercise, on menstrual pain. Researchers should explore other factors, such as hormonal imbalances or stress, to see how they contribute to the severity of dysmenorrhea. Studies could test new ways to manage or prevent menstrual pain, such as alternative therapies, to help reduce its impact on women's daily lives.

## Conclusions

Primary dysmenorrhea is a prevalent condition among female students at Umm Al-Qura University and has a profound impact on their quality of life. The physical, emotional, and academic disruptions caused by menstrual pain highlight the need for improved awareness, management strategies, and support systems for affected individuals. By addressing the multifaceted nature of dysmenorrhea and considering both genetic and lifestyle factors, healthcare providers can offer more comprehensive care to young women experiencing this condition. Future research should continue to explore effective interventions and preventive measures to reduce the burden of dysmenorrhea on women's health and daily lives.
